# Resolutions of Respect to Dr. Charles A. Kingsbury

**Published:** 1892-04

**Authors:** L. Ashley Faught

**Affiliations:** Secretary


### Obituary.

### RESOLUTIONS OF RESPECT TO DR. CHARLES A. KINGSBURY.

THE following resolutions were passed at the last meeting of the Board of Directors of the Philadelphia County Dental Society:

WHEREAS, It has pleased our Heavenly Father to call to rest Dr. Charles A. Kingsbury, our friend, associate, active member, and one of the founders of this Society, therefore, be it

*Resolved,* That we desire to express our high esteem for one whose faithful and earnest work is widely known throughout the profession, and

*Resolved,* That this record of our deep regret be entered upon the minutes of this Society, and a copy of these resolutions be sent to the dental journals for publication.

L. ASHLEY FAUGHT,
*Secretary.*

